# Data in risk assessment of mega-city infrastructures related to land subsidence using improved trapezoidal FAHP

**DOI:** 10.1016/j.dib.2019.105007

**Published:** 2019-12-17

**Authors:** Hai-Min Lyu, Shui-Long Shen, Annan Zhou, Jun Yang

**Affiliations:** a Key Laboratory of Intelligent Manufacturing Technology (Shantou University), Ministry of Education, Shantou, Guangdong 515063, China; b Department of Civil and Environmental Engineering, College of Engineering, Shantou University, Shantou, Guangdong 515063, China; c Civil and Infrastructure Engineering Discipline, School of Engineering, Royal Melbourne Institute of Technology (RMIT), Victoria 3001, Australia; d Department of Civil Engineering, The University of Hong Kong, Pokfulam, Hong Kong, China

**Keywords:** Land subsidence, Risk assessment, Trapezoidal fuzzy number, FAHP, GIS

## Abstract

Land subsidence caused serious damages of mage-city infrastructures. This data in brief presents a new questionnaire to establish judgment matrix during the risk assessment of land subsidence. The data source of the assessment factors is provided. The analytical hierarchy process (AHP) and interval fuzzy AHP (FAHP) are used to calibrate the weights of assessment factors. The new questionnaire is used to collect the viewpoints from experts. Based on the viewpoints of experts, the judgment matrix can be established using pairwise comparison. The data presented herein was used for the article, titled “Risk assessment of mega-city infrastructures related to land subsidence using improved trapezoidal FAHP” Lyu et al. (2019) [1].

**Table:** Specifications Table

Subject area	Engineering
More specific subject area	Safety, Risk, Reliability and Quality
Type of data	Table
How data was acquired	The assessment data was obtained from official internet sites of public administration and statistics. Part of the data was obtained through an expert survey on the importance degree between the influencing factors and risks.
Data format	Raw, analyzed
Experimental factors	The data were processed with 30 m resolution in GIS before analysis.
Experimental features	The data were collected from the website of local government and the statistic yearbook of Shanghai (see Table 2).
Data source location	Shanghai, China
Data accessibility	Data are included in this article
Related research article	Lyu, H.M., Shen, S.L., Zhou, A.N., Yang, J. Risk assessment of mega-city infrastructures related to land subsidence using improved trapezoidal FAHP, Science of the Total Environment, published online: https://doi.org/10.1016/j.scitotenv.2019.135310

**Table:** 

**Value of the Data** • The data sources of all assessment factors related to the research article [1] are provided. • The data article provides a new questionnaire, which is used to collect viewpoints from experts. • Based on the viewpoints from the new questionnaire, the judgment matrix with the trapezoidal fuzzy number can be established. • The data article provides a calculation process to determine the trapezoidal fuzzy number and then establish the fuzzy judgment matrix, which can aid researchers and analysts in understanding how to apply the trapezoidal FAHP with the new questionnaire. • The new questionnaire can be applied in other cases related to risk assessment.

## 1 Data

Data including the hazard and vulnerability is used to assess the risk induced by land subsidence to significant infrastructures. Table 1 lists the data source and description of the assessment factors. Table 2 lists the vulnerability index for the risk assessment of the land subsidence [2,3]. Based on the obtained assessment factors, both the traditional and new questionnaires were used to obtain the viewpoints of the experts. Table 3 presents the new questionnaire. Table 4 comprises the linguistic variables and corresponding trapezoidal fuzzy number. The trapezoidal fuzzy number is used to express the importance of the assessment factors. Table 5 presents the statistical viewpoints obtained from six experts. Table 6 presents the extended trapezoidal FAHP judgement matrix for the hazard index. Table 7 presents the extended trapezoidal FAHP judgement matrix for the vulnerable index.

**Table 1:** Data sources and description of each factor.

Index	Sub-index	Description	Data source and format
*H*_*i*_	*H*_1_	Hazard intensity of land subsidence	Data from Shanghai Institute of Land Resource Survey
	*H*_2_	Groundwater extraction intensity	
	*H*_3_	Historical land subsidence	
	*H*_4_	Historical settlement rate	
	*H*_5_	Potential land subsidence	Author's research result with 30 m resolution
	*H*_6_	Average ground elevation	Geospatial data cloud with 30 m resolution
*V*_*j*_	*V*_1_	Population density	Data from reference SSB (2017) [2]
	*V*_2_	Gross domestic product (GDP) per unit area	
	*V*_3_	Construction land ratio	
	*V*_4_	Metro line density	
	*V*_5_	Industrial output per unit area	
	*V*_6_	Elevated road density	
	*V*_7_	Disaster reduction input	
	*V*_8_	Recharge groundwater input

**Table 2:** Data for vulnerability index assessment of Shanghai land subsidence division district (Data from SSY, 2017).

District	*V*_1_ (×10^3^p/km^2^)	*V*_2_ (billion/km^2^)	*V*_3_ (%)	*V*_4_ (km/km^2^)	*V*_5_ (billion/km^2^)	*V*_6_ (km/km^2^)	*V*_7_ (×10^3^ rmb/km^2^)	*V*_8_ (×10^3^ rmb/km^2^)
Urban centre	24.07	2.51	93.47	1.03	7.48	1.59	363.8	2861.4
Pudong	4.55	3.7	70.48	0.43	7.67	0.44	192.5	128.4
Minhang	6.85	1.24	70.33	0.32	8.50	0.2	72.2	7.0
Jiading	3.40	0.89	47.77	0.12	11.54	0.03	84.2	91.5
Baoshan	7.49	1.07	67.46	0.28	6.61	0.4	230.9	159.0
Songjiang	2.91	0.75	40.36	0.09	5.52	0	20.1	1.5
Jinshan	1.37	0.33	35.3	0	2.67	0	47.2	7.9
Qingpu	1.81	0.46	29.31	0	2.31	0	30.7	2.1
Fengxian	1.70	0.32	26.69	0	2.08	0	31.6	10.6
Chongming	0.59	0.07	11.18	0	0.30	0	37.5	44.9

**Table 3:**
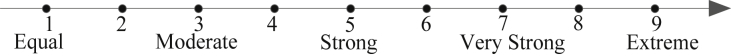
Newly designed consulting questionnaire for the risk assessment of land subsidence.

Factor	Influence of the factor on the risk induced by land subsidence								
	1	2	3	4	5	6	7	8	9
Factor 1									
Factor 2									
Factor 3									
Factor 4									
……									
Factor n									

Note: to ensure that each score can be assigned, you are suggested to assign each score to no more than two factors. Please tick [✓] in any one rating that you feel is appropriate for each factor.

**Table 4:** Linguistic variables and corresponding trapezoidal fuzzy number.

Linguistic terms	Ordinary assignment (AHP)	Trapezoidal fuzzy number
Equal	1	1′= (1,1,1,1)
Slightly strong	3	3′= (1,1.222,1.857,2.333)
Fairly strong	5	5′= (1.5,1.857,3,4)
Very strong	7	7′= (2.333,3,5.667,9)
Absolutely strong	9	9′= (4,5.667,9,9)

(2,4,6,8) and (2′,4′,6′,8′) imply that the importance degrees belong to the interval variables.

**Table 5:** Statistical viewpoints from six experts.

Factor	Influence of the factor on the risk induced by land subsidence								
	1	2	3	4	5	6	7	8	9
Hazard intensity of land subsidence (*H*_1_)							I	I	IV
Groundwater extraction intensity (*H*_2_)							II	II	II
Historical land subsidence (*H*_3_)				II		III	I		
Historical settlement rate (*H*_4_)				I	III	II			
Potential land subsidence (*H*_5_)			I	III	II				
Average ground elevation (*H*_6_)	II	III	I						
Population density (*V*_1_)								IV	II
GDP per unit area (*V*_2_)					II	II	II		
Construction area ratio (*V*_3_)				I	II	II			I
Metro system density (*V*_4_)					I	II	I	II	
Industrial output per unit area (*V*_5_)						I	I	IV	
Elevated road density (*V*_6_)						I	II	II	I
Disaster reduction input (*V*_7_)	I				II		II	I	
Recharge groundwater input (*V*_8_)		II	II		II				

Note: Roman number in table represents selected times of the score from 1 to 9.

**Table 6:** Extended trapezoidal FAHP judgement matrix for hazard index.

	*H*_1_	*H*_2_	*H*_3_	*H*_4_	*H*_5_	*H*_6_
*H*_1_	(1,1,1,1)	(1,1,1,1)	(1,1.111,1.429,1.667)	(1,1.222,1.857,2.333)	(1,1.111,1.429,1.667)	(1,1.111,1.429,1.667)
*H*_2_	(1,1,1,1)	(1,1,1,1)	(1,1.111,1.429,1.667)	(1,1.111,1.429,1.667)	(1,1.111,1.429,1.667)	(1,1.111,1.429,1.667)
*H*_3_	(0.6,0.7,0.9,1)	(0.6,0.7,0.9,1)	(1,1,1,1)	(1,1.222,1.857,2.333)	(1,1.222,1.857,2.333)	(1,1.111,1.429,1.667)
*H*_4_	(0.429,0.538,0.818,1)	(0.6,0.7,0.9,1)	(0.429,0.538,0.818,1)	(1,1,1,1)	(1.5,1.857,3,4)	(1.917,2.429,4.334,6.5)
*H*_5_	(0.6,0.7,0.9,1)	(0.6,0.7,0.9,1)	(0.429,0.538,0.818,1)	(0.25,0.333,0.538,0.667)	(1,1,1,1)	(1,1.222,1.857,2.333)
*H*_6_	(0.6,0.7,0.9,1)	(0.6,0.7,0.9,1)	(0.6,0.7,0.9,1)	(0.154,0.231,0.412,0.522)	(0.429,0.538,0.818,1)	(1,1,1,1)

**Table 7:** Extended trapezoidal FAHP judgement matrix for vulnerability index.

	*V*_1_	*V*_2_	*V*_3_	*V*_4_	*V*_5_	*V*_6_	*V*_7_	*V*_8_
*V*_1_	(1,1,1,1)	(1,1,1,1)	(1,1.111,1.429,1.667)	(1,1.222,1.857,2.333)	(1.25,1.540,2.429,3.167)	(1.5,1.857,3,4)	(1.5,1.857,3,4)	(1.5,1.857,3,4)
*V*_2_	(1,1,1,1)	(1,1,1,1)	(1,1,1,1)	(1,1.111,1.429,1.667)	(1,1.222,1.857,2.333)	(1.25,1.540,2.429,3.167)	(1.5,1.857,3,4)	(1.5,1.857,3,4)
*V*_3_	(0.6,0.7,0.9,1)	(1,1,1,1)	(1,1,1,1)	(1,1.111,1.429,1.667)	(1,1.111,1.428,1.667)	(1,1.222,1.857,2.333)	(1,1.222,1.857,2.333)	(1.25,1.540,2.429,3.167)
*V*_4_	(0.429,0.538, 0.818,1)	(0.6,0.7,0.9,1)	(0.6,0.7,0.9,1)	(1,1,1,1)	(1,1.111,1.428,1.667)	(1,1.222,1.857,2.333)	(1.25,1.540,2.429, 3.167)	(1.25,1.540,2.429,3.167)
*V*_5_	(0.316,0.412, 0.649,0.8)	(0.429,0.538,0.818,1)	(0.6,0.7,0.9,1)	(0.6,0.7,0.9,1)	(1,1,1,1)	(1,1.222,1.857,2.333)	(1.25,1.540,2.429, 3.167)	(1.5,1.857,3,4)
*V*_6_	(0.25,0.333,0.538,0.667)	(0.316,0.412,0.649,0.8)	(0.6,0.7,0.9,1)	(0.6,0.7,0.9,1)	(0.6,0.7,0.9,1)	(1,1,1,1)	(1,1.111,1.428,1.667)	(1,1.111,1.428,1.667)
*V*_7_	(0.25,0.333,0.538,0.667)	(0.25,0.333, 0.538,0.667)	(0.429,0.538,0.818,1)	(0.316,0.412,0.649, 0.8)	(0.316,0.412,0.649,0.8)	(0.6,0.7,0.9,1)	(1,1,1,1)	(1,1,1,1)
*V*_8_	(0.25,0.333,0.538,0.667)	(0.25,0.333,0.538,0.667)	(0.316,0.412, 0.649,0.8)	(0.316,0.412,0.649, 0.8)	(0.25,0.333,0.538,0.667)	(0.6,0.7,0.9,1)	(1,1,1,1)	(1,1,1,1)

## 2 Experimental design, materials and methods

### 2.1 Consulting questionnaire

**Fig. 1:**
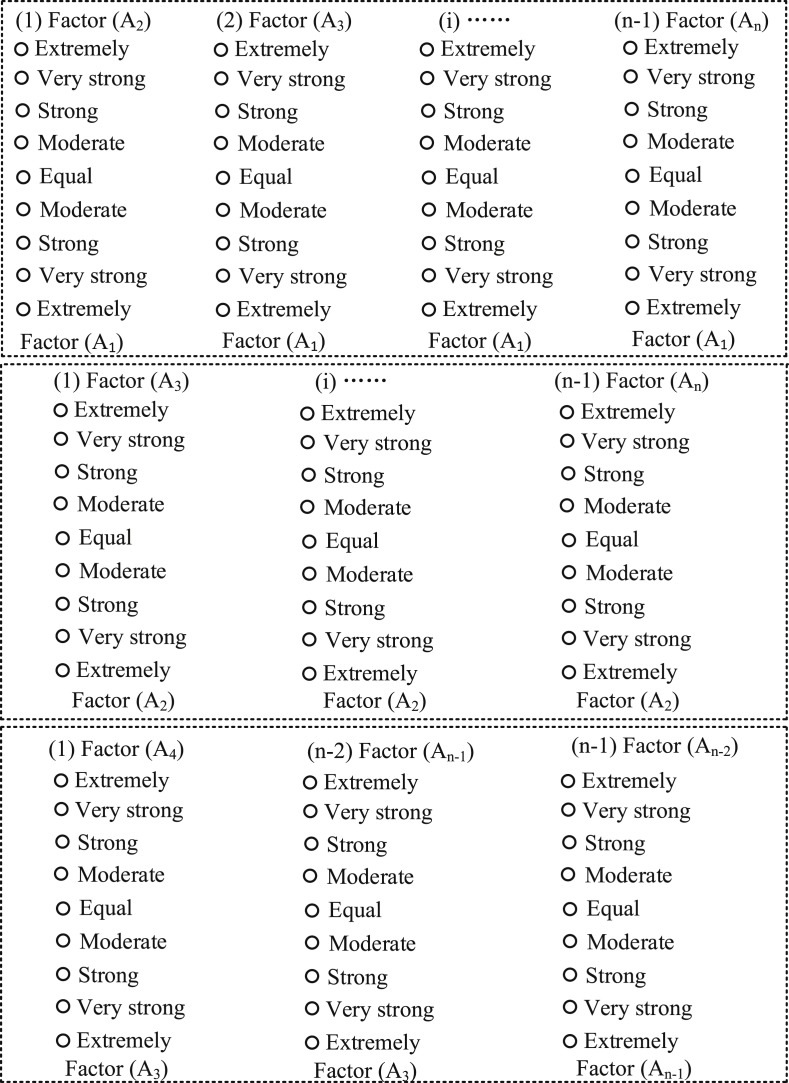
Traditional questionnaire for pairwise comparison.

Fig. 1 shows the traditional questionnaire. Pairwise comparisons were used in the traditional questionnaire. In the traditional questionnaire, each assessment factor is compared with another [4,5]. The traditional questionnaire has two limitations: (i) obtaining expert judgments using the traditional questionnaire is tedious and time-consuming, and (ii) inconsistencies frequently arise from subjective expert judgments, which produces an inconsistent judgment matrix [6,7]. Assuming that there are *n* factors, every expert can make a number of pairwise comparisons *n*(*n*-1)/2 (see Fig. 1). The total number of pairwise comparisons increases when multiple factors are involved in the risk assessment hierarchy. The new questionnaire comprises the use of nine scores for obtaining the viewpoints of the experts (Table 3). The experts are required to assign a score to a factor. Based on the expert responses obtained using the new questionnaire, in the next analysis step, the analysts can make pairwise comparisons and establish a consistent judgment matrix [8,9]. Based on the consistent judgment matrix and the score obtained using the new questionnaire, the analysts can determine the triangular fuzzy numbers according to Table 4. Finally, the fuzzy judgment matrix can be established.

### 2.2 Responses from new questionnaire

Table 5 lists the statistical viewpoints from six experts. As listed in Table 5, the score for *H*_1_ ranges from 7 to 9; therefore, *H*_1_ is initially assigned as 7–9. It is noteworthy that 9 is selected four times. Owing to the same reason, *H*_2_ = 7–9, considering that both 7 and 9 are selected twice; *H*_3_ = 4–7, with 4 selected twice and 6 thrice; *H*_4_ = 4–6, with 5 selected thrice and 6 twice; *H*_5_ = 3–5, with 4 selected thrice and 5 twice; *H*_6_ = 1–3, with 2 selected thrice and 1 twice. Each element in the judgement matrix can be expressed as a ratio of one interval number to another, such as $\frac{H_{1}}{H_{2}}=\frac{7-9}{7-9}$, $\frac{H_{1}}{H_{3}}=\frac{7-9}{4-7}$, $\frac{H_{1}}{H_{4}}=\frac{7-9}{4-6}$, $\frac{H_{1}}{H_{5}}=\frac{7-9}{3-5}$, $\frac{H_{1}}{H_{6}}=\frac{7-9}{1-3}$, etc. Thus, a pairwise comparison judgement matrix can be obtained. Similarly, the judgment matrix of vulnerability index can also be obtained [[10], [11], [12]].

### 2.3 Establishment of trapezoidal fuzzy judgment matrix

Once the judgment from the Table 5 demand the consistent requirement, the trapezoidal fuzzy judgment can be established by replacing the trapezoidal fuzzy number (see Table 5). In the replacement process of each factor, it is noteworthy that the selection time of each score was considered to construct the triangular fuzzy number to obtain a trapezoidal fuzzy number that is as close as possible to the original ratio. Table 6, Table 7 list the judgement matrices with trapezoidal fuzzy numbers. The detailed calculation process can refer the related companion article Lyu et al. [1].
